# Supplementary material for: A Dual Biomarker TK1 Protein and CA125 or HE4-Based Algorithm as a Better Diagnostic Tool than ROMA Index in Early Detection of Ovarian Cancer
Source: Cancers (Basel). 2023 Mar 3;15(5):1593. doi: 10.3390/cancers15051593 (PMC10000714; doi:10.3390/cancers15051593)
Supplement: Supplementary file 1 [file cancers-15-01593-s001.zip › cancers-2176861-supplementary.pdf]

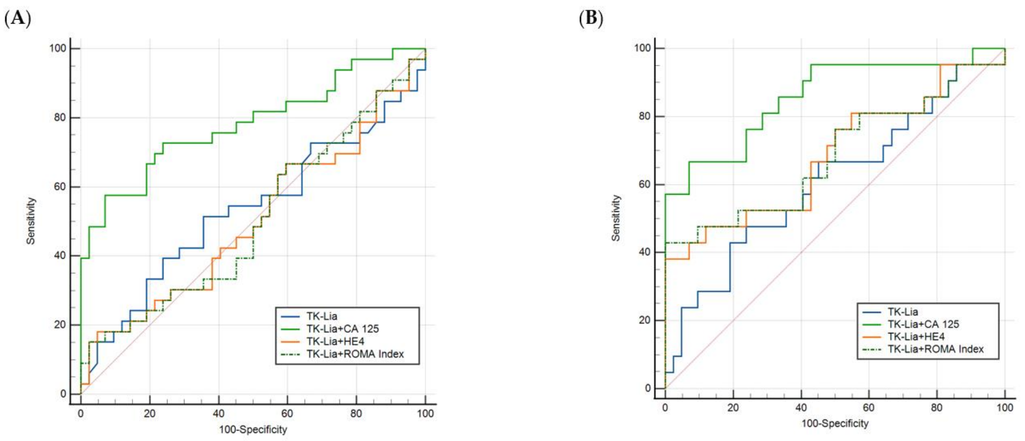

Figure S1. (A,B). Roc curves for TK-Liaison assay in combination with other biomarkers in the differentiation of premenopausal benign and malignant from controls.

Table S1. ROC curve analysis for TK-Liaison assay in combination with other biomarkers in the differentiation of premenopausal benign and malignant from controls.

**Premenopausal**						
Benign vs controls	Cut-off	AUC	Sensitivity	Specificity	PPV	NPV
TK-Liaison	9.2	0.533	15.2	95.2	71.4	58.8
TK-Lia+ CA 125	0.68	0.784	48.5	97.6	94.1	70.7
TK-Lia+HE4	0.47	0.503	18.2	95.2	75.0	59.7
TK-Lia+ROMA Index	0.51	0.504	15.1	97.6	83.3	59.4
Malignant vs controls						
TK-Liaison	9.2	0.61	23.8	95.2	71.4	71.4
TK-Lia+ CA 125	0.64	0.858	57.1	100	100	82.4
TK-Lia+HE4	0.48	0.685	38.2	100	100	76.4
TK-Lia+ROMA Index	0.49	0.685	42.8	100	100	77.8
